# Information Theory for Agents in Artificial Intelligence, Psychology, and Economics

**DOI:** 10.3390/e23030310

**Published:** 2021-03-06

**Authors:** Michael S. Harré

**Affiliations:** Complex Systems Research Group, Faculty of Engineering, The University of Sydney, Sydney 2006, Australia; michael.harre@sydney.edu.au

**Keywords:** decision theory, psychology, artificial intelligence, economics, artificial neural networks, neuroscience, information theory, Bayesian learning, free energy

## Abstract

This review looks at some of the central relationships between artificial intelligence, psychology, and economics through the lens of information theory, specifically focusing on formal models of decision-theory. In doing so we look at a particular approach that each field has adopted and how information theory has informed the development of the ideas of each field. A key theme is expected utility theory, its connection to information theory, the Bayesian approach to decision-making and forms of (bounded) rationality. What emerges from this review is a broadly unified formal perspective derived from three very different starting points that reflect the unique principles of each field. Each of the three approaches reviewed can, in principle at least, be implemented in a computational model in such a way that, with sufficient computational power, they could be compared with human abilities in complex tasks. However, a central critique that can be applied to all three approaches was first put forward by Savage in *The Foundations of Statistics* and recently brought to the fore by the economist Binmore: Bayesian approaches to decision-making work in what Savage called ‘small worlds’ but cannot work in ‘large worlds’. This point, in various different guises, is central to some of the current debates about the power of artificial intelligence and its relationship to human-like learning and decision-making. Recent work on artificial intelligence has gone some way to bridging this gap but significant questions remain to be answered in all three fields in order to make progress in producing realistic models of human decision-making in the real world in which we live in.

## 1. Introduction

**Figure 1:**
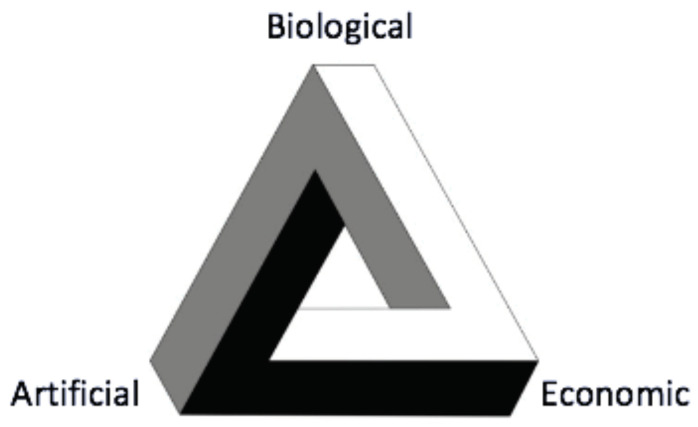
A schematic of the relationships between the three fields. Each element has a direct link of the same colour to the other two, but no single path navigates the whole circuit.

The goal of this article is to review a relatively unexplored but interesting intersection between three different research areas that motivated the special issue this article appears in: economics, artificial intelligence, and psychology. One way in which these fields intersect with one another is via decision theory, each bringing something different to the discussion but with points of tension in how they conceptualise the issues. In highlighting a subset of the differences and similarities, this article aims to provide a perspective that is not well covered in the literature. To do this we review these fields from an information theoretical perspective, chosen because of its growing popularity as an empirical tool, but also because it has noteworthy properties in the context of decision theory, providing a overarching technical framework. Each field also has a relatively well understood path to the other two, but seen as a whole there is a tension between all three that suggests further exploration, Figure 1 illustrates this unification-tension in decision theory. In this Introduction we very briefly cover some earlier uses of information theory in each field, and more details are also given in the following sections, before describing how the article is structured. It is hoped that those interested in recent progress in these three fields, how they overlap with one another, and the theoretical concerns in implementing them in simulations will find this article of interest.

One of the most important contributors to the debate regarding entropy and its use in economics comes from the work Georgescu-Roegen [1]. In their extensive body of work Georgescu-Roegen was concerned with the analysis of an entire economic process and the relationship it has with entropy and thermodynamics. At the smaller scale of markets and in the economics of population dynamics, Wilson’s entropic methods [2] have been used to study long term population flows using methods based on maximum entropy [3] and Fisher information [4]. The stability of financial markets have been studied using mutual information [5,6] and transfer entropy [7], drawing an analogy from the instability of aggregate economic dynamics and the physics of phase-transitions [8] in which mutual information [9], transfer entropy [10], and Fisher information [11] have been used to examine strongly non-linear systems. In market dynamics, Aoki [12] has used maximum entropy methods to model the approach to stationarity including interactions between agents and these same maximum entropy techniques have been used to studied game theory [13,14]. However, despite these diverse applications and both the necessary software [15] and texts introducing the methods [16] (particularly Chapter 6 on finance and economics) there has only been limited take-up in mainstream economics.

In psychology, the history of information theory is also quite uneven and Laming has written an extensive history and critique [17] of the use of these methods in psychology. Early on the application of Shannon’s ideas to psychology were enthusiastic, shortly after the development of information theory in 1948 [18] Miller published their 1956 article *The magical number seven, plus or minus two: Some limits on our capacity for processing information* [19]. In this well-cited article information theory is used to measure *the span of absolute judgement*, what might be called our short-term or working memory today, estimating it to contain around seven items. At about the same time (1954) McGill published their article *Multivariate information transmission* [20] using sophisticated formal methods to analyse multivariate data, stating:

It is not widely understood, however, that the tools made available by communication theory are useful for analyzing data, whether or not we believe that the human organism is best described as a communications system.

So these two papers lay the foundations for psychology to use information theory as both a method for data analysis and as a method for understanding human cognition as information storage or processing. However, as the mathematical psychologist Duncan Luce has written [21], information theory has never played a central role in psychology. Luce’s article is critical of the information theory - psychology partnership, in writing on the psychological studies of mean response times to stimuli, a common experimental paradigm still used today, Luce writes ([21] p. 185):

In early experiments, mean response time appeared to grow linearly with uncertainty, but glitches soon became evident. The deepest push in the response time direction was Donald Laming’s (1968) subtle *Information Theory of Choice-Reaction Times*, although he later stated: “This idea does not work. While my own data (1968) might suggest otherwise, there are further unpublished results that show it to be hopeless” (Laming, 2001, p. 642).

The enthusiasm—nay, faddishness—of the times is hard to capture now. Many felt that a very deep truth of the mind had been uncovered. Yet, Shannon was skeptical [...] Myron Tribus (1979, p. 1) wrote: “In 1961 Professor Shannon, in a private conversation, made it quite clear to me that he considered applications of his work to problems outside of communication theory to be suspect and he did not attach fundamental significance to them.” These skeptical views strike me as appropriate.

Luce sees the key issue to be in the hidden structure of human responses to stimuli, but information theory, he argues, is agnostic to these subtleties of cognitively mediated relationships between stimuli. For example, he describes the highly sensitive nature of the correlation structure of human response times when there is either short or long separations between the stimuli, implying that they are not separable in a way that information theory requires them to be ([21] p. 185):

However, in my opinion, the most important answer lies in the following incompatibility between psychology and information theory. The elements of choice in information theory are absolutely neutral and lack any internal structure; the probabilities are on a pure, unstructured set whose elements are functionally interchangeable.

However some recent work in the expertise literature has been able to show that, at least at an aggregate data-analysis level, some of the results obtained in the psychology of decision theory have been re-cast in an information theoretical framework [22,23,24], matching the earlier results that used more conventional methods. In these studies some of the high level qualitative features observed in human behavioural studies can be seen using information theory, suggesting that the data-analytical use of information theory has applications in studies of behavioural psychology. In what follows we will see that, at least to some extent, these concerns are not as insurmountable as Luce or Laming suggest, but that we are also still a long way away from being able to draw definitive conclusions.

Within the field of neuroscience though there has been much greater take-up of information theoretical methods, of which only two are mentioned here. In network inference and data analysis the development of software such JIDT [15] and IDTxL [25] that can be applied to neuro-physiological data [26,27,28] has made the application of computationally intensive, information theoretical tools much easier in recent years. In another specialisation, theoretical work on decision theory and cognition [29,30,31,32] has made significant inroads in modelling some of the theoretical principles that underpin decision-making, and the neuro-scientific principles of learning, memory, and behaviour that connect these theories to practical concerns have begun to emerge [33,34].

Finally, in the field of artificial intelligence (AI) methods based on information theory such as mutual information and relative (cross) entropy have had a long and productive history [35]. However, a distinction should be made between different perspectives regarding the purpose of studying artificial intelligence. For example, as a model of human cognition there are a number of quite different mathematical approaches at different levels of analysis and development such as the free energy principle [36] (which is the focus of Section 5), integrated information theory [37], or the AIXI agent for universal intelligence [38]. Each of these approaches attempts to formally describe human intelligence and is less concerned with, although not totally disinterested in, the consequences for AI applied to data analysis.

On the other hand there is an extensive body of work that uses AI as a tool for the analysis of large and complex data-sets and these are less concerned with what this tells us about human cognition and decision-making. However these methods often make use of ideas from information theory in order to improve performance, for example in feature selection tasks mutual information is used to measure the relevance of different features [39] as well as feature selection using entropy-based filters [40], information gain methods [41], one-shot learning [42], and deep learning [43]. In these applications information theory plays an important role, but the emphasis is towards the practical implementation of effective algorithms for data analysis, rather than the foundations of decision-making.

There is a third position between these two ends of the scale that is also worth mentioning because these AIs are opening up the debate on the potential for AI to perform at human like levels on tasks that are complex enough to challenge humans in ways that are quite surprising, albeit still in relatively narrow domains. A candidate for the earliest of the most recent evolution of “big data” AI to challenge human-like reasoning is IBM’s Watson [44], an AI that in 2012 was able to win against past human champions of the US game show *Jeopardy!*, a game where the contestants need to guess the question given the answer, a type of backward induction that requires sophisticated aspects of inference and contextualisation that humans are particularly good at and AIs were particularly poor at. The next candidate is AlphaGo [45] that beat the 9 dan Go champion Lee Sedol in 2016 and its decedent, AlphaZero [46], that learned to play Go, Chess, and Shogi entirely from self-play. The last example is the auto-regressive language model called GPT-3 [47], probably the most controversial of these three AIs because of its ability to generate natural language that can be mistaken for human creative writing and reasoning. For a brief introduction see the OpenAI API website (https://openai.com/blog/openai-api/ (accessed on 9 May 2015)) and for a review of GPT-3’s ability to ‘think’ by forming analogies see [48]. These AIs have generated considerable debate as researchers and practitioners work through what is hyperbole and what is reasonable extrapolation of the near future of AI and it’s ability to perform human-like reasoning, for a recent review of what currently seems plausible in AI and its shortcomings see [49].

In the following Sections we introduce some of the historical background for each field and then focus on a very specific line of research or reasoning in order to illustrate the broader perspective of this article. The reason is a matter of practicality: each field could not be summarised very easily in a single article, although where I know of such an article I have referenced it, and to attempt a full review for all three fields is probably beyond any reasonable length article. So I have selected work that has particularly motivated this special issue, and so it is naturally idiosyncratic for that reason, but there are some principles I have roughly followed to further narrow the field. The work needs to be well-established with substantial prior work to draw on and ample opportunity for it to have been critiqued. It also needs to be expressible formally so that the mathematical connections, if they exist, can be discussed. The final section brings some of the similarities and differences into play in order to illustrate where the lines of tension lie and what this may mean for future work. There are many other ways to have sliced and diced these topics, but I hope the mix that appears here provides an interesting and illuminating perspective.

## 2. Economic Rationality and Expected Utility

What we mean by “rationality” and what it means for how we make decisions has a long and extensively debated past, see for example the many different definitions that can be found in [50]. The historical background to these debates is important for understanding the different interpretations of rationality, but for this section economic rationality will mean *rational decision theory* using *expected utility theory* that is summarised in [51] and follows Binmore’s approach [52] that is based on Savage’s original work on Bayesian decision theory [53]. In this section, I introduce the axioms of economic decision theory in order to make clear exactly what utility means in economics and how it is interpreted in the mainstream (classical) sense. The key point is that this interpretation of rationality avoids the need to have any representation of the psychological processes that result in a decision being made.

An agent is given a set of lotteries L for which each $l_{i}\in L$ is a random process with *j* possible outcomes $o_{i,j}$ that occur with probability $p_{i,j}$. We can also combine lotteries in the following way. Say the agent is given two lotteries to choose between, $l_{1}$ with probability *p* and $l_{2}$ with probability 1−p, then we can define a third lottery as the combination of these two lotteries: $\left\{pl_{1},\left(1-p\right)l_{2}\right\}$. We also use the symbol ⪯ to represent a weak preference relationship between two lotteries $l_{1}$ and $l_{2}$ from a set L. We interpret $l_{1}⪯l_{2}$ as meaning that $l_{2}$ is at least as preferable as $l_{1}$, and the agent can then weakly order the lotteries in L. With these definitions we can state the necessary axioms regarding lotteries and preferences that are used in establishing Savage’s key theorems:

**Axiom** **1.** *Completeness:*

*For any $l_{1}$, $l_{2}$∈L either $l_{1}⪯l_{2}$ or $l_{2}⪯l_{1}$.*

**Axiom** **2.** *Transitivity:*

*For any $l_{1}$, $l_{2}$, $l_{3}$∈L, if $l_{1}⪯l_{2}$ and $l_{2}⪯l_{3}$, then $l_{1}⪯l_{3}$.*

In the case where $l_{1}⪯l_{2}$ and $l_{2}⪯l_{1}$ an agent is indifferent to these two lotteries and we use the notation: $l_{1}∼l_{2}$. The last two axioms are:

**Axiom** **3.** *Continuity:*

*Suppose we have $l_{1}$, $l_{2}$∈L, then for any p∈[0,1] there is a third lottery $l_{3}$ such that: $\left\{pl_{1},\left(1-p\right)l_{2}\right\}∼l_{3}$.*

**Axiom** **4.** *Independence:*

*Suppose we have $l_{1}$, $l_{2}$∈L, then for any $l_{3}$ and p∈[0,1]: $\left\{pl_{1},\left(1-p\right)l_{3}\right\}⪯\left\{pl_{2},\left(1-p\right)l_{3}\right\}$.*

These axioms are sufficient to state and prove one of the key theorems of economic decision theory:

**Theorem** **1.** ***Savage** ([53] Thm. 1 p. 73 and Thm. 4. p. 75)*

*If an agent is asked to choose between two lotteries $l_{i},l_{j}\in L$ and their behaviour satisfies Axioms 1–4 above then there exists real-valued utilities $U(l_{i})$ such that:*

(1) $$\begin{array}{ccc} l_{i}⪯l_{j} & \Leftrightarrow & U\left(l_{i}\right)\leq U\left(l_{j}\right) \end{array}$$

That is to say that a weakly ordered preference over choices that has been observed in the behaviour of an agent, and without reference to the inner workings of the agent’s mind, is sufficient to infer a numerical utility function. Finally, choosing a lottery is *rational*, or the agent is said to act rationally if the agent chooses the lottery $l_{*}$ that would be consistent with maximising their expected utility:

(2) $$\begin{array}{ccc} l_{*} & = & \max_{i}U\left(l_{i}\right)\;\;\forall \;\;l_{i}\in L \end{array}$$

This axiomatic approach has played a fundamental role in expected utility theory, but equally important is the subsequent relationship between *utility* and *rationality* that informs the interpretation of economic rationality in the context of psychological or cognitive processes.

Binmore has written that economic theory does not make any claims about the *cognitive processes* that maximise a utility function, and goes further to say that within economics a utility function does not need to exist at all. The following passage is taken from their book *Rational Choice*:

Economists after Bentham [1748–1832] became increasingly uncomfortable, not only with the naïve hypothesis that our brains are machines for generating utility, but with all attempts to base economics on psychological foundations. The theory of revealed preference therefore makes a virtue of assuming nothing whatever about the psychological causes of our choice behavior [...] Modern decision theory succeeds in accommodating the infinite variety of the human race within a single theory simply by denying itself the luxury of speculating about what is going on inside someone’s head. Instead, it pays attention only to what people do. ([52] Chapter 1: Revealed Preferences)

This argument is based on the idea that agents behave *as if* they were being driven by a psychological process that is using an expected utility function. The *as if* argument was introduced in a highly influential article by Milton Friedman on “Positive Economics” [54] and by now the argument has a long history, see for example the recent collection of articles in [55]. Binmore articulated the consequences of *as if* reasoning for revealed preferences in *Rational Choice*:

In revealed-preference theory, it isn’t true that [an agent] chooses *b* rather than *a* because the utility of *b* exceeds the utility of *a*. This is the Causal Utility Fallacy. It isn’t even true that [an agent] chooses *b* rather than *a* because she prefers *b* to *a*. On the contrary, it is because [an agent] chooses *b* rather than *a* that we say that [they] prefer *b* to *a*, and assign *b* a larger utility. ([52] Chapter 1: Revealed Preferences)

To summarise this view:

The rationality of expected utility theory requires an agent’s preferences to be described by Axioms 1–4, that they are stable over time, and that they are revealed through the agent’s behaviour. From these premises an observer can infer, by Savage’s theorems, that an agent’s preferences can be represented using a numerical utility function in the form of Equation (1) and that the agent’s behaviour is optimal with respect to this inferred utility function. However this does not mean that a utility function exists or is used by the agent to optimise their decision, optimisation is inferred by consistency of preferences, and the agent only behaves as though a utility exists and as though they are using it to optimise the choices they make.

The history of the wide acceptance of expected utility theory in economics and the role of Savage, Friedamn, and Samuelson is an interesting story of how the theoretical difficulties that Samuelson saw in expected utility theory were resolved by introducing Axiom 4 (Independence), finally winning Samuelson over to the theory [56]. From expected utility theory Savage showed how an economic agent can use Bayes’ theorem (see Section 4) to update their probabilities when new information arrives, see ([52] Chapter 7) for a review of Savage’s analysis in this context. In the next section we extend these ideas to a recent approach to decision theory and information theory.

## 3. Free Energy

At this point we have yet to connect economic decisions with information theory. To move in that direction we introduce work that was developed using ideas from physics in such a way that statistical uncertainty in the choice an agent will make connects the *optimal* decisions described in the previous section with *sub-optimal* decisions and consequently information theory. The central idea from physics is that of the *free energy*.

### 3.1. Free Energy in Physics

We first introduce the Helmholtz free energy $F_{p}$ used in physics as the internal energy of the system V(x) having discrete states *x* less the Shannon entropy of the system:

(3) $$\begin{array}{ccc} H(x) & = & -\sum_{i}P\left(x_{i}\right)\log\left(P\left(x_{i}\right)\right) \end{array}$$

multiplied by the temperature of the system *T*:

(4) $$\begin{array}{ccc} F_{p} & = & V(x)-TH(x). \end{array}$$

This is the energy that is available to do work in a thermodynamic system at equilibrium. It removes the energy that is not available due to thermal fluctuations: the smaller the *T* value and consequently the smaller the thermal fluctuations are, the smaller the contribution from the entropy becomes, and so more of the total energy of the system *V* is available for doing thermodynamic work.

Equation (4) has an analogue in neuroscience that is the basis of what Friston calls the Free Energy Principle of the brain, but before describing that, we briefly introduce the same general principles in an economic context.

### 3.2. Free Utility in Economics

Equation (4) has an economic counterpart that appears, for example, in the earlier work of Wolpert [57,58] on predictive game theory and collective intelligence. In its simplest form an agent chooses a distribution *p* over the utilities $U(x_{i})$ over a finite set of discrete choices $\left\{x_{i}\right\}$ such that they have an expected utility:

(5) $$\begin{array}{c} E_{p}\left[U\left(x\right)\right]=\sum_{i}p_{i}U\left(x_{i}\right) \end{array}$$

The ‘free utility’ of this situation is given by:

(6) $$\begin{array}{ccc} F_{g} & = & E_{q}\left[U\left(x\right)\right]-TH\left(x\right), \end{array}$$

in which *T* is analogous to the thermodynamic temperature and represents a decision-maker’s uncertainty in their choice: the lower their uncertainty (small *T*) the higher their free utility, and the entropy measures the utility lost due to the sub-optimality of their choice. In a game theory setting, Equation (6) can be derived from an exponential distribution of probabilities over choices that can be found by using Jaynes’ method of maximising the entropy where the expected utility of the game is the constraint [13,14,59], this results in the well known quantal response equilibrium of McKelvey and Palfrey [60]. This is true in general where we might know, for example through experimental observation, that an economic problem has a constraint in the form of an expectation: $E_{p}\left[G\left(x\right)\right]$ and we would like to know the “least informative” [61] distribution that satisfies this constraint. Then Jaynes’ MaxEnt [62] provides a way of deriving the following distribution:

(7) $$\begin{array}{ccc} P(x_{i}) & = & Z^{-1}e^{\beta E_{p}\left[G\left(x\right)\right]} \end{array}$$

with Z normalising the distribution and the free energy is:

(8) $$\begin{array}{ccc} F(p) & = & \frac{\partial Z}{\partial \beta }+\beta^{-1}\sum_{x}P\left(x\right)\log\left(P\left(x\right)\right), \end{array}$$

(9) $$\begin{array}{ccc} & = & E_{p}\left[G\left(x\right)\right]-\beta^{-1}H\left(x\right),\\ & = & \mathrm{Utility}-\beta^{-1}\mathrm{Entropy}. \end{array}$$

If F(p) is then minimised over the distribution p(x) we will arrive back at Equation (7).

A similar approach was taken in the work of Grünwald and Dawid on Bayes optimal decisions and game theory [63]. There they sought to generalise constrained maximum entropy methods by casting MaxEnt as minimising a log-loss function and then generalising the method to other loss functions. Amongst others, the following results were established:

- Using MaxEnt to form constrained distributions over decision variables is compatible with Bayes optimal decisions,
- It brings maximising the entropy into correspondence with maximising the expected utility when the utility is a log-loss function,
- These methods can be generalised to finding other optimal, constrained strategies using a variety of loss functions.

These results illustrate that there is an underlying discipline-independent aspect to optimisation in decision theory that respects Savage’s Bayesian principles in economics and it can be framed in terms of entropic methods. In the next section we turn to the approach developed by Jaynes to model a robot decision-maker, a prototype of an idealised AI.

## 4. The Logic of Jaynes’ Learning Robot

In *Probability Theory: The Logic of Science* [64] Jaynes introduces a robot (generalised AI) as a rational decision-maker that follows certain desiderata that reflect rationality. For a history of the use of Bayes theory, going as far back as Herodotus and the necessity of reasoning, Jaynes has written an extensive review [65] that covers a broader context of learning, inference, and probability theory. For their own contributions in *Probability Theory*, he introduces their robot in the following way:

In order to direct attention to constructive things and away from controversial irrelevancies, we shall invent an imaginary being. Its brain is to be designed by us, so that it reasons according to certain definite rules. These rules will be deduced from simple desiderata which, it appears to us, would be desirable in human brains; i.e., we think that a rational person, should he discover that he was violating one of these desiderata, would wish to revise their thinking ([64] Chapter 1: The thinking computer)

The desiderata that are going to be built into the robot are in three parts ([64] Chapter 1: Plausible reasoning): 

Desideratum (I):

Degrees of Plausibility are represented by real numbers. Desideratum (I) is practically forced on us by the requirement that the robot’s brain must operate by the carrying out of some definite physical process. However, it will appear that it is also required theoretically; we do not see the possibility of any consistent theory without a property that is equivalent functionally to Desideratum (I).

Desideratum (II):

Qualitative Correspondence with common sense. We want to give our robot another desirable property for which honest people strive without always attaining; that it always reasons consistently.

Desideratum (IIIa):

If a conclusion can be reasoned out in more than one way, then every possible way must lead to the same result.

Desideratum (IIIb):

The robot always takes into account all of the evidence it has relevant to a question. It does not arbitrarily ignore some of the information, basing its conclusions only on what remains. In other words, the robot is completely non-ideological.

Desideratum (IIIc):

The robot always represents equivalent states of knowledge by equivalent plausibility assignments. That is, if in two problems the robot’s state of knowledge is the same (except perhaps for the labeling of the propositions), then it must assign the same plausibilities in both.

Jaynes identifies these desiderata with how their rational robot should reason. Specifically, their robot uses the following fundamental principle to inform all probabilistic inferences that it makes:

*To form a judgement about the likely truth or falsity of any proposition A, the correct procedure is to calculate the probability that A is true:*

(10) $$\begin{array}{c} P(A|E_{1}E_{2}\ldots ) \end{array}$$

*conditional on all the evidence at hand.* ([64] p. 86)

Jaynes goes on to establish how the robot uses Bayes’ theorem to learn and reason. He begins with a list of various propositions denoted *A*, *B*, *C*, etc., letting AB stand for the proposition “both *A* and *B* are true”, $\bar{A}$ for “*A* is false”, and P(A|B) represents the probability that *A* is true given *B*, Jaynes restates the product and sum rules of probability:

(11) $$\begin{array}{ccc} P(AB|C) & = & P(A|BC)P(B|C) \end{array}$$

(12) $$\begin{array}{ccc} 1 & = & P\left(A|B\right)+P\left(\bar{A}|B\right) \end{array}$$

(13) $$\begin{array}{ccc} P(A|BC) & = & P\left(A|C\right)\frac{P(B|AC)}{P(B|C)} \end{array}$$

where the last statement is Bayes’ theorem. For Jaynes, the key point embedded in Bayes’ theorem is the functional ability for their robot to be able to learn from new information. To see this consider:

(14) $$\begin{array}{ccc} P(DH|X) & = & P(H|DX)P(D|X)\\ X & = & \mathrm{prior}\mathrm{information}\\ H & = & \mathrm{an}\mathrm{hypothesis}\mathrm{to}\mathrm{test}\\ D & = & \mathrm{the}\mathrm{observed}\mathrm{data}\\ \Rightarrow P(H|DX) & = & P\left(H|X\right)\frac{P(D|HX)}{P(D|X)} \end{array}$$

where the *prior probability* P(H|X) is updated with new information *D* to give the *posterior probability* P(H|DX), and Equation (13) provides Jaynes’ reasoning robot with the computational steps to do so.

This is only the first part of the reasoning that the robot can do though. In ([64] Chapter 11) Jaynes extends these foundations to how a robot might reason in a more versatile fashion, pointing out that the robot does not yet have a way of assigning probabilities to prior information at the outset of a problem. According to Jaynes initially all the robot knows how to do is to break the problem up into a set of exhaustive, mutually exclusive states and assign a uniform probability based on the principle of indifference. However, if there is some prior information that can be be expressed as an expectation then there is a principled method by which the robot can use this information as *mean valued constraints* in order to establish a Bayesian prior from which the robot can begin to learn and reason. This is formally the same as the method Jaynes established in physics [62], i.e., it is precisely the maximum entropy method of statistical mechanics but expanded and applied to the robot’s decision-making processes.

By now it has been established in the literature that Bayesian methods and maximum entropy methods are consistent with respect to each other and they both satisfy some criteria that various school’s of thought agree a reasonable decision-maker should respect [63]. In particular, Grünwald and Dawid established the formal relationship between what had, prior to their work, been suspected but not entirely formalised: that there is “a close relationship between two problems that have customarily been regarded as distinct: that of maximizing entropy, and that of minimizing worst-case expected loss [...] these two problems are shown to be dual to each other, the solution to each providing that to the other.”

With that in mind, there is a close and self-consistent relationship between maximising the free utility in game theory, maximising entropy subject to mean valued constraints, and Bayesian decision theory. In the following section some of these elements come together in the free energy principle that Friston has proposed for modelling human cognition in a naturalistic environment.

## 5. Friston’s Free Energy Principle

In order to introduce the free energy principle without the complexity of Friston’s complete model, a simpler procedural framework is introduced first before introducing the complete distributions with more realistic inputs, this follows [66]. We start with an agent who knows that there is a joint distribution P(s,o) that describes the relationship between sensory observations *o* and the real states of the world *s* (hidden to the agent) that generated them. This joint distribution can be decomposed into a conditional probability and a marginal probability: P(o,s)=P(o|s)P(s), i.e., the Bayesian likelihood and prior, respectively. P(o,s) is called the generative model and is described in more detail below. In Bayesian cognitive science, the generative model is said to be inverted to give the posterior distribution P(s|o)∝P(o|s)P(s) also called the recognition model.

The difficulty with the direct application of this method is that the likelihood is often computationally expensive or even intractable for all but the simplest models. To address this we can estimate the posterior with an approximate posterior (described in detail below): Q(s)≃P(s|o), i.e., the new recognition model Q(s) is an approximation to the Bayesian posterior. In order to compute Q(s) we minimise the free energy:

(15) $$\begin{array}{ccc} Q(s) & = & \arg\min_{Q}F\left(Q\right)\;\;\Rightarrow \;\;Q\left(s\right)\;\;≃P\left(s|0\right) \end{array}$$

(16) $$\begin{array}{ccc} F(Q) & = & E_{Q}\left[\log\left(Q\left(s\right)\right)-\log\left(P\left(s|o\right)\right)\right] \end{array}$$

(17) $$\begin{array}{ccc} & = & E_{Q}\left[-\log\left(P\left(s|o\right)\right)\right]-H\left(Q\left(s\right)\right) \end{array}$$

which converts the problem of having to directly estimate the likelihood to a more tractable optimisation problem. This is functionally the same as the free utility of an expected log-loss function −log(P(s|o)) where expectation is over Q(s), minus the entropy of Q(s).

Friston’s wider framework expands on the structural form of the distributions just described. In order to do this we introduce the following discrete sets [67] where the *external world* is the environment outside of the agent’s cognitive process, i.e., it is demarcated by the agent’s sensory organs communicating states of the world to the agent’s internal cognitive processes:

- A finite set of observations Ω about the external world.
- A finite set of actions *A* the agent can take in the external world.
- A finite set of hidden states *S* of the external world.
- A finite set of control states *U* that the agent can choose from in order to act in the external world.

With these sets we can describe the three probability distributions that are used in Friston’s approach to describing an adaptive agent interacting with their environment.

**Distribution** **1.** *The first distribution is the generative process that produces an agent’s observations $o_{i}\in \Omega$ over a discretely indexed period of time t∈{0,…,T} such that o(t)∈Ω and $\tilde{o}=\left\{o\left(0\right),\ldots ,o\left(T\right)\right\}$ is a specific instance of a time series of o(t) observations. This notation is used for the other state variables $s_{i}\in S$, $a_{i}\in A$, and $u_{i}\in U$ as well. With variables s(t)∈S and a(t)∈A, the generative process is defined as the joint probability distribution:*

(18) $$\begin{array}{ccc} R(\tilde{o},\tilde{s},\tilde{a}) & = & P(\left\{o\left(0\right),\ldots ,o\left(T\right)\right\}=\tilde{o},\left\{s\left(0\right),\ldots ,s\left(T\right)\right\}=\tilde{s},\left\{a\left(0\right),\ldots ,a\left(T\right)\right\}=\tilde{a}). \end{array}$$

**Distribution** **2.** *The generative model over observations is a belief model that the agent has regarding how the external world generates the agent’s observations. Given a time series of observations o(t)∈Ω, of hidden states s(t)∈S, and of control states u(t)∈U the generative model is:*

(19) $$\begin{array}{ccc} P(\tilde{o},\tilde{s},\tilde{u}\;|\;m) & = & P(\left\{o\left(0\right),\ldots ,o\left(T\right)\right\}=\tilde{o},\left\{u\left(0\right),\ldots ,u\left(T\right)\right\}=\tilde{u},\left\{s\left(0\right),\ldots ,s\left(T\right)\right\}=\tilde{s}), \end{array}$$

(20) $$\begin{array}{ccc} & = & P\left(\tilde{o}|\tilde{s}\right)P\left(\tilde{s},\tilde{u}|\gamma ,\tilde{a}\right)P\left(\gamma |m\right) \end{array}$$

*in which $P(\tilde{o}|\tilde{s})$ is the likelihood of the sequence of observations $\tilde{o}$ given the hidden sequence of states $\tilde{s}$ and:*

(21) $$\begin{array}{ccc} P(\tilde{s},\tilde{u}|\gamma ,\tilde{a}) & = & P\left(\tilde{u}|s\left(t\right),\gamma \right)\;\;P\left(s\left(t\right)|s\left(t-1\right),a\left(t-1\right)\right)\ldots P\left(s\left(1\right)|s\left(0\right),a\left(0\right)\right)\;\;P\left(s\left(0\right)|m\right) \end{array}$$

*is the agent’s beliefs about the state transitions of the Markovian process that generates the next hidden state(s) of the world from the previous state and the agent’s previous action: P(x(t)|x(t−1)s(t−1)). P(γ|m) is the likelihood of the parameters γ given the agent’s model m of the external world. The utility is derived from the first term in Equation (21):*

(22) $$\begin{array}{ccc} \log(P\left(\tilde{u}|s\left(t\right),\gamma \right)) & = & \beta U_{tot}\left(\tilde{u},s\left(t\right)\right) \end{array}$$

(23) $$\begin{array}{ccc} U_{tot}\left(\tilde{u},s\left(t\right)\right) & = & H\left(P\left(s\left(T\right)|s\left(t\right),\tilde{u}\right)\right)+\sum -P\left(s\left(T\right)|s\left(t\right),\tilde{u}\right)\log\left(P\left(s\left(T\right)|m\right)\right) \end{array}$$

*In this expression the first term is the entropy and represents the utility associated with an agent’s exploration of states and the second term is the expected utility of a log-loss function for the distribution P(s(T)|m), which is just the expectation of the log-probability of the external world’s final state s(T) given the agent’s priors encoding their goals m.*

**Distribution** **3.** *The last distribution is the approximate posterior distribution over the hidden and control states:*

(24) $$\begin{array}{ccc} Q(\tilde{s},\tilde{u}) & = & P(\left\{s\left(0\right),\ldots ,s\left(T\right)\right\}=\tilde{s},\left\{u\left(0\right),\ldots ,u\left(T\right)\right\}=\tilde{u}). \end{array}$$

*This is the approximate posterior distribution of Equation (15) that is needed to be found through the free energy optimisation process.*

**The free energy expression:** Substituting the last two distributions into Equation (17) we have the following form:

(25) $$\begin{array}{ccc} F(Q\left(\tilde{s},\tilde{u}\right)) & = & E_{Q}\left[-\log\left(P\left(\tilde{o},\tilde{s},\tilde{u}\;|\;m\right)\right)\right]-H\left(Q\left(\tilde{s},\tilde{u}\right)\right), \end{array}$$

(26) $$\begin{array}{ccc} \Rightarrow Q(\tilde{s},\tilde{u}) & = & \arg\min_{Q}F\left(Q\left(\tilde{s},\tilde{u}\right)\right). \end{array}$$

This is the final form of Friston’s free energy principle for how an agent estimates the posterior joint distribution of $Q(\tilde{s},\tilde{u})$, i.e., by optimising in order to find the (approximate) Bayesian posterior distribution, the agent is implicitly maximising a utility function that is embedded in the second term of the generative model given by Equation (20). The utility is explicitly expressed in Equation (23), which in turn was derived from the first term of Equation (21). This is achieved by the agent manipulating the control states $\tilde{u}$ that maximise the expected utility of the final state of the external world s(T).

## 6. Collecting the Threads

What I have sought to illustrate with the discussion above are the three different perspectives each has adopted, and how they have been framed in different ways by different researchers, but the underlying formalisms are not very far apart. For example, axiomatic decision theory has had a powerful influence on how we understand what we should want or expect from an ideal decision-maker, and once decision-theory is framed in terms of an ‘ideal’ these axioms are very appealing. However, axiomatic methods do not necessarily reflect what happens under the cognitive hood of our psychology and so they can only be part of the solution to understanding how real agents make decisions, for example in new environmental contexts. Psychology and neuroscience take the opposite position to the axiomatic approach in saying that the cognitive process is the point at which investigation begins, and if a cohesive theory ultimately comes from that then all the better, but the empirical foundations are central. Finally, while AI looks under the hood to some extent, it also has a bi-modal approach: one mode emphasising the optimality of a data-intensive analysis, the other emphasising the implementation of some decision-making desiderata, not unlike economic decision-theory.

Each of these approaches can, in principle, be implemented in software or hardware, if only because they are describable mathematically, and in fact they were included here for this very reason. This might not get us any closer to realising the true potential of these algorithms to model human-like decision-making for many decades due to a lack of computational power, but the potential may already be there. This would still leave us with the question of how much of our human abilities need to be retained in models of decision theory for a particular application, for example in a macro-economic model we might only need a very simple model of each agent but to realistically model two people playing a game like Go or chess we likely need to model most of a person’s neuro-psychology such as perception, long term memory, and detailed strategising and mind-reading (in the psychological *theory of mind* sense).

Before moving on to the main parts of this section it is important to note two particularly illustrative directions in artificial intelligence that have extended our understanding of what is theoretically and practically possible. Successfully playing Go against a professional player was considered at least a decade away when Lee Sedol was defeated in 2016, and that this was a grand challenge for AI, and since then the AlphaGo ecosystem of AIs has grown and been successful in other areas that were considered to be too challenging for current AI as well. However, getting to the moon cannot be achieved by technological breakthroughs in ladder design, and it is not clear whether our current AI technology is just a “ladder” or an under-powered “rocket” in terms of human intelligence and decision-making, and this debate has not yet played itself out by any means, see [49] for a wide-ranging view of the current limitations of AI. What is clear though is that there is likely to be a place for both rockets and ladders of many different designs in the future applications of AI. In another direction in AI, Pearl has carried out extensive work on Bayesian networks and their applications to artificial intelligence and decision-theory [68,69]. The importance of this work lies in the potential for Bayesian belief networks to encode human-like expertise in a rigorous and implementable fashion. Of particular interest in the context of this review is that Pearl’s work [70] has informed aspects of Da Costa and Friston’s et al. work on probabilistic graphical models for active inference [71]. Much like the results discussed above for artificial intelligence, this is very recent work and it looks to be a very promising direction for further work. In the next subsections I discuss some potential directions for applying information theory to decision theory.

### 6.1. Do Not Look under the Hood!

Expected utility theory has been the dominant paradigm in economic decision theory at least since the capitulation of Samuelson in 1950 [56], and despite recent challenges from alternatives such as behavioural economics [72], prospect theory [73], and maxmin expected utility theory [74] it still remains central to economic theory [56]. The theory is motivated by the principle that agents make choices *as if* they were maximising a utility function and that an economist can infer the existence of this function through the observation of an agent’s preferences. In this frame the economist chooses not to look under the hood [75] of the agent making a decision, i.e., they deliberately choose not to understand psychological processes. This is a form of *substantive rationality*, *what* decisions are made is more important than *how* they are made [76]. In this setting an agent is rational if their *behaviour* is appropriate for the achievement of their goals (such as maximising an inferred utility function) within the conditions and constraints of their environment [77]. Simon summarises the point here:

Thus, the assumptions of utility or profit maximization, on the one hand, and the assumption of substantive rationality, on the other, freed economics from any dependence upon psychology. ([77] p. 66)

Taking this view at face value, we ask: what can information theory contribute to economics in this mode? Recall from the Introduction that McGill proposed information theory as an instrument for investigating multivariate time series [20] where the patterns of observed behaviour of the agents are encoded in the data. These patterns, assuming that they are individually identified, can be treated as the *revealed preferences* of individual agents. There are some caveats that would need to be carefully considered, for example the complete set of choices available to each agent might not be known to the economist or the agent, and the environment and constraints might be non-stationary etc. but these are issues for any analysis of this type. Now suppose we want to know how each agent’s behaviour influences the others, a common question in game theory but also of interest more generally in studies of social and economic interactions. Expected utility theory provides no guidance on the nature of an agent’s utility function and it might be linear or non-linear. Traditional methods such as the Pearson correlation can measure the linear correspondence between the patterns of behaviour of two agents, but if the patterns are nonlinear they can be missed entirely by conventional techniques while mutual information, for example, will measure both the linear and non-linear aspects of behavioural correspondences [78,79,80]. Furthermore, modern software can be extended to the analysis of discrete event data of a continuous time process with limited modifications [7], allowing researchers to infer temporally directed relationships between events, even in complex time series.

What we conclude is that, even if we choose not to look under the psychological hood, information theory is a much more sensitive instrument for measuring non-linearities in behaviour, and therefore non-linear utilities, see for example [81].

### 6.2. OK, Should We Look under the Hood Anyway?

The alternative to *substantive rationality* is *procedural rationality* [77] in which we are concerned with *how* an agent makes a decision, rather than *what* decision they make. Hausman gives the following argument equivalent to Friedman’s original *as if* logic in order to critique Friedman’s *Positive Economics* ([75] p. 184):

1. A good used car drives safely, economically, and comfortably. (over-simplified premise)
2. The only test of whether a used car is a good used car is whether it drives safely, economically, and comfortably. (invalidly from 1)
3. Anything one discovers by opening the hood and checking the separate components of a used car is irrelevant to its assessment. (trivially from 2)

So what is wrong with this argument? Hausman argues that fundamentally the future behaviour (performance) of the car cannot be reasonably, i.e., within cost and time bounds, assessed by simply taking it for a drive. Opening the hood and looking at the condition of the engine, looking at the chassis, and the current state of all the other components of the car will give a much clearer picture of what the car will cost for repair and maintenance into the future, whether or not it will breakdown in the next six months, and every used car buyer would be sensible to do such tests.

This analogy shares a great deal with another analogy, this one used by one of the founders of complexity economics, Brian Arthur [82], about the flow of traffic on a freeway (an interview: W. Brian Arthur on “Complexity Economics” https://www.complexityexplorer.org/news/19-w-brian-arthur-on-complexity-economics (accessed on 9 May 2015)). In this analogy traffic can be modelled as a steady state equilibrium of traffic flows for much of the time, but traffic jams form and they do not stem from just one car or from every car, the jam is caused by a small number of cars and their interactions (for example the distance between them, breaking and accelerating times). So in order to understand the complex patterns that emerge in traffic flows we need to look beyond equilibrium behaviour and understand the meso-scopic properties that cause these complexities to emerge.

Similarly for our decision-makers, if we choose to look under the psychological hood we need to find the right scale of analysis. Friston’s free energy approach is interesting in this respect because it has both neuronal and psychological [83] explanatory power. In terms of what information theory may say about this approach, there is an important result that states, for a suitably defined utility function U(.) and any probability space (Ω,F,P) we have that: U(A|B)=f(P(A|B)) and f(.)=αlog(.) (α>0) from which the conclusion follows that utility *is* information in the form of (scaled) surprisal, see [31] and references therein. While this is in agreement with the Friston model as well as the Grünwald and Dawid methods, it approaches the problem from a different direction and makes explicit that the log-loss function is the same as utility, and that expected utility is the expected log-loss.

So a key strength in looking under the hood is that we get a much richer view of the generative processes of behaviour than we would if all we knew was the current repertoire of behaviours in a static world of stationary preferences. This has an impact when for example new technologies become available or when an equilibrium state is disturbed, two facets of modern life that are becoming more and more prominent as our everyday experience is disrupted by events that are difficult to foresee based on forecasting from past events. In this way we can use simulations based on the underlying cognitive processes to test and make predictions about an agent’s behaviour in circumstances that have not yet been observed.

### 6.3. How Large Is Our World?

To date, it might still seem plausible that a clever combination of ‘big data’, massive computing power, and some Bayesian brain modelling using neural networks might be enough to reasonably model human decision making, even if we need to wait a few decades for the necessary technology to make it practical. If this were true then, in terms of the theory (if not the hardware and software), we would essentially be done. This is the central concern of Binmore’s book [52] as well as a central point of Savage’s original thesis [53]: that Bayesianism is not always rational and when it is not rational is precisely where we fail to understand the difference between our theories of decision-making and how decision-making biological agents operate in the real world.

Savage ([53] p. 16) described a ‘small world’ as being the situation in which a decision-maker can always “look before you leap”, i.e., all information about the consequences of each choice is available to the decision-maker. A ‘large world’ is the situation where “you can cross the bridge when you come to it”, i.e., the consequences are not known before a decision is made, so the decision-maker will deal with them as they arise. For example, game theory is a small world problem, but the financial markets are not. Binmore identifies the work of Gilboa and Schmeidler [84] as being a case in point where economics has attempted to tackle this issue by studying imitation as an aspect of rationality. This approach was the inspiration for an artificial neural network approach to expert-level understanding of the patterns in the game of Go [23,24], a situation that is very much in the ‘large world’ setting.

The issue that Savage and Binmore raise is both subtle and key to decision theory in each of the contexts covered earlier in this article. Binmore has recently written a summary of the problems he sees in the construction of Bayesian priors [85]:

There is no consensus on where such a logical prior comes from, but scholars who see Bayesianism as embodying the essence of rational learning usually argue that we should place the choice of prior at a time in the past when Alice is completely ignorant. An appeal is then sometimes made to the “Harsanyi doctrine”, which says that different rational agents independently placed in a situation of complete ignorance will necessarily formulate the same common prior (Harsanyi 1977). However, even if one accepts this doubtful principle, one is left wondering what this “rational prior” would look like.

In practice, some version of Laplace’s principle of insufficient reason is usually employed. Others take this position further by arguing that the complete ignorance assumption implies, for example, that the rational prior will maximize entropy (Jaynes and Bretthorst 2003). We are then as far from Savage’s view on constructing priors as it is possible to be. Instead of using all potentially available information in the small world to be studied in formulating a prior, one treats all such information as irrelevant.

The literature offers many examples that draw attention to the difficulties implicit in appeals to versions of Laplace’s principle. Similar objections can be made to any proposal for choosing a logical prior that depends only on the state space. Even in small worlds we seldom know the “correct” state space. In a large world, we have to update our state space as we go along—just as physicists are trying to do right now as they seek to create a theory of everything.

In brief, Bayesianism has no solid answer to the question of where logical priors come from. Attempts to apply versions of the principle of insufficient reason fail because they take for granted that the state space is somehow known a priori beyond any need for revision. The same goes for any proposal that makes the prior a function of the state space alone. However, how can Alice be so certain about the state space when she is assumed to know nothing whatever about how likely different states may be?

As mentioned above, Jaynes answer to this is to say that prior information for their robot can be used by encoding it as an expectation and this, via MaxEnt, leads to a well defined statistical model that can then be updated through Bayesian learning. This still does not address the concerns Binmore raises, but it appears to take the debate one step closer to a practical resolution, if not a theoretical one that reflects an axiomatic approach to decision theory. Friston also addresses this issue for the free energy principle here:

Kersten et al. (2004) provide an excellent review of object perception as Bayesian inference and ask a fundamental question, ‘Where do the priors come from. Without direct input, how does image-independent knowledge of the world get put into the visual system?’ In §3(e), we answer this question and show how empirical Bayes allows priors to be learned and induced online during inference. [29]

So let us compare what Friston says in their §3(e) with what Binomore has to say about massaging information in order to get consistent priors, Friston’s central point is this:

Empirical Bayes allows these priors to be induced by sensory input, using hierarchies of backward and lateral projections that prevail in the real brain. In short, hierarchical models of representational learning are a natural choice for understanding real functional architectures and, critically, confer a necessary role on backward connections.

This pivots on the method of *empirical Bayes*, an approach to establishing priors directly from data in a hierarchical structure:

The problem with fully Bayesian inference is that the brain cannot construct the prior expectation and variability [...] *de novo*. They have to be learned and also adapted to the current experiential context. This is a solved problem in statistics and calls for empirical Bayes, in which priors are estimated from data. Empirical Bayes harnesses the hierarchical structure of a generative model, treating the estimates at one level as priors on the subordinate level (Efron & Morris 1973). This provides a natural framework within which to treat cortical hierarchies in the brain, each level providing constraints on the level below. This approach models the world as a hierarchy of systems where supraordinate causes induce and moderate changes in subordinate causes. These priors offer contextual guidance towards the most likely cause of the input.

In contrast, Binmore’s appeal to ‘massaging’ the priors:

At the end of the story, the situation is as envisaged by Bayesianites: the final “massaged” posteriors can indeed be formally deduced from a final “massaged” prior using Bayes’ rule. This conclusion is guaranteed by the use of a complex adjustment process that operates until consistency is achieved. [...]

Notice that it is not true in this story that [an agent] is learning when the massaged prior is updated to yield a massaged prior - that rational learning consists of no more than applying Bayes rule. On the contrary, Bayesian updating only takes place after all learning is over. [...] Bayesianites therefore have the cart before the horse. Insofar as learning consists of deducing one set of beliefs from another, it is the massaged prior that is deduced from the unmassaged posteriors [86].

Binmore’s first paragraph could be in a agreement with Friston’s use of hierarchical methods from empirical Bayes, but the second paragraph makes it clear that Binmore does not see any room in ‘Bayesianism’ for empirical learning. What Binmore appears to be looking for in the second paragraph is the learning that occurs before any Bayesian updating or massaging occurs, but this is what Friston appears to be offering in their neural framework of a cortical hierarchy, although it is not clear from reading their respective views how either would respond without explicitly putting the case to each of them. Another intriguing point is that Friston’s view appears to be compatible with a perceptual deep-learning AI with feed-forward feed-back mechanisms, and in that sense the Large World of Savage and Binmore may well be (partially) addressed by these early sensory information processing mechanisms. This may well be an approach to understanding how the AlphaGo AI is able to perform so well in the Large(-ish) World of Go: it is able to build priors dynamically and from scratch, further complicating the issue around the capabilities of AI’ approach to theoretical limits in decision-making. Note that Savage and Binmore may consider Go to be a Large World, or at least larger than a Small World, but it is still very small relative to the real world and these AIs are still highly dependent on operating within the boundaries of their task environment.

### 6.4. Where to for the Future?

There are many reasons for exploring these different approaches and how they intersect with one another, two possibilities are mentioned here. The first is that AI could be used as a model of human reasoning to help us better understand and improve upon our socioeconomic systems [87]. We can see that some of our greatest risks are instabilities caused by interactions between opposing views in politics, economics, and other spheres of our lives, so can better models of how we make decisions individually and collectively help address these issues? This is a grand challenge but psychologically well-founded models of people’s behaviour could help us understand how behaviour at the individual level causes patterns of instability at larger social scales. The second is that, as Brian Arthur has observed [82], the US (and the world) economy is becoming increasingly more autonomous, but can AI be used to supplement, rather than supplant, people in the workplace to make working conditions better? This needs highly sophisticated AIs that use accurate models of human psychology in order to interact with people effectively. However, the challenges are exceptional, AI has gone some small way to looking under the psychological hood and the small world of Savage has been made a little larger with recent advances on more complex tasks. However, AIs are still prone to failure in only slightly modified environments that people would find trivial to navigate and they do not yet have the resilience and adaptability people have in making a decision. A particular hurdle is that, in each of the examples in this article, the agents do not appear to have a way to *understand* a decision-making task or to be able to work effectively when they cannot look before they leap. Melanie Mitchell, in *Artificial intelligence hits the barrier of meaning* [49] sums up the challenge for these areas in the future:

It may be that the pure information processing metaphor is not sufficient, and that other frameworks might give the insights that we need. Some proposals for such frameworks include free energy principles, control theory, dynamical systems theory, and ideas from biological development. It may be that an entirely new framework is needed for a full account of cognition.

## Data Availability

This study used no data.
